# Sensitization of TRPV1 by EP_1 _and IP reveals peripheral nociceptive mechanism of prostaglandins

**DOI:** 10.1186/1744-8069-1-3

**Published:** 2005-01-17

**Authors:** Tomoko Moriyama, Tomohiro Higashi, Kazuya Togashi, Tohko Iida, Eri Segi, Yukihiko Sugimoto, Tomoko Tominaga, Shuh Narumiya, Makoto Tominaga

**Affiliations:** 1 Dept.of Cellular and Molecular Physiology, Mie University School of Medicine, Mie 514-8507, Japan; 2 Okazaki Institute for Integrative Bioscience, National Institutes of Natural Sciences, Okazaki 444-8787, Japan; 3 Department of Physiological Sciences, The Graduate University for Advanced Studies, Okazaki 444-8787, Japan; 4 Dept. ofPharmacology, Kyoto University Faculty of Medicine, Kyoto 606-8501, Japan; 5 Dept. of Physiological Chemistry, Graduated School of Pharmaceutical Sciences, Kyoto University, Kyoto 606-8501, Japan

## Abstract

Prostaglandin E_2 _(PGE_2_) and prostaglandin I_2 _(PGI_2_) are major inflammatory mediators that play important roles in pain sensation and hyperalgesia. The role of their receptors (EP and IP, respectively) in inflammation has been well documented, although the EP receptor subtypes involved in this process and the underlying cellular mechanisms remain to be elucidated. The capsaicin receptor TRPV1 is a nonselective cation channel expressed in sensory neurons and activated by various noxious stimuli. TRPV1 has been reported to be critical for inflammatory pain mediated through PKA- and PKC-dependent pathways. PGE_2 _or PGI_2_increased or sensitized TRPV1 responses through EP_1 _or IP receptors, respectively predominantly in a PKC-dependent manner in both HEK293 cells expressing TRPV1 and mouse DRG neurons. In the presence of PGE_2 _or PGI_2_, the temperature threshold for TRPV1 activation was reduced below 35°C, so that temperatures near body temperature are sufficient to activate TRPV1. A PKA-dependent pathway was also involved in the potentiation of TRPV1 through EP_4 _and IP receptors upon exposure to PGE_2 _and PGI_2_, respectively. Both PGE_2_-induced thermal hyperalgesia and inflammatory nociceptive responses were diminished in TRPV1-deficient mice and EP_1_-deficient mice. IP receptor involvement was also demonstrated using TRPV1-deficient mice and IP-deficient mice. Thus, the potentiation or sensitization of TRPV1 activity through EP_1 _or IP activation might be one important mechanism underlying the peripheral nociceptive actions of PGE_2 _or PGI_2_.

## Background

Tissue damage and inflammation produce an array of chemical mediators such as ATP, bradykinin, prostanoids, protons, cytokines and peptides including substance P that can excite or sensitize nociceptors to elicit pain at the site of injury. Among them prostanoids were shown to influence inflammation, and their administration was found to reproduce the major signs of inflammation including augmented pain [1]. Prostaglandin E_2 _(PGE_2_) and prostaglandin I_2 _(PGI_2_) are the products of arachidonic acid metabolism through the cyclooxygenase pathway. In addition to numerous other physiological actions *in vivo*, previous studies have indicated important roles for PGE_2 _in nociception and inflammation [2,3]. PGE_2 _is generated in most cells in response to mechanical, thermal or chemical injury and inflammatory insult, resulting in sensitization or direct activation of nearby sensory nerve endings. Analgesic effects of non-steroidal anti-inflammatory drugs (NSAIDs) are attributed predominantly to inhibition of prostaglandin synthesis. Prostaglandins act upon a family of pharmacologically distinct prostanoid receptors including EP_1_, EP_2_, EP_3_, EP_4 _and IP that activate several different G protein-coupled signaling pathways [2,4,5]. Primary sensory neurons in dorsal root ganglion (DRG) are known to express mRNAs encoding several prostanoid receptor subtypes, IP, EP_1_, EP_3 _and EP_4 _[6,7]. The role of IP in inflammation has been clearly shown by the analysis of IP-deficient mice, although the underlying cellular mechanisms still remain to be elucidated [8]. In contrast, the potential involvement of EP receptors other than IP in inflammation and pain generation has not been well studied, although some earlier studies have suggested that prostanoids contribute to the development of pain through EP receptors [9,10].

The capsaicin receptor TRPV1 is a non-selective cation channel expressed predominantly in unmyelinated C-fibers [11]. TRPV1 is activated not only by capsaicin, but also by protons or heat (with a threshold > ~43°C), both of which cause pain *in vivo *[11-13]. A prominent role of TRPV1 in nociception has been demonstrated in studies of TRPV1-deficient mice [14,15].

Recently, we reported that inflammatory mediators such as ATP, bradykinin and trypsin or tryptase potentiate TRPV1 activity in a PKC-dependent manner [16-18], and identified two target serine residues in TRPV1 as substrates for PKC-dependent phosphorylation [19]. On the other hand, there are several reports showing that a PKA signaling pathway mediates PGE_2_-induced potentiation of capsaicin-evoked responses in rat sensory neurons [20-22]. Therefore, we examined the effects of PGE_2 _and PGI_2 _on TRPV1 activity. Surprisingly, we found the functional interaction of TRPV1 with PGE_2 _or PGI_2 _occurs mainly through a PKC-dependent pathway at both cellular and behavioral levels.

## Results

### Functional interaction between TRPV1 and PGE_2_

**Figure 1:**
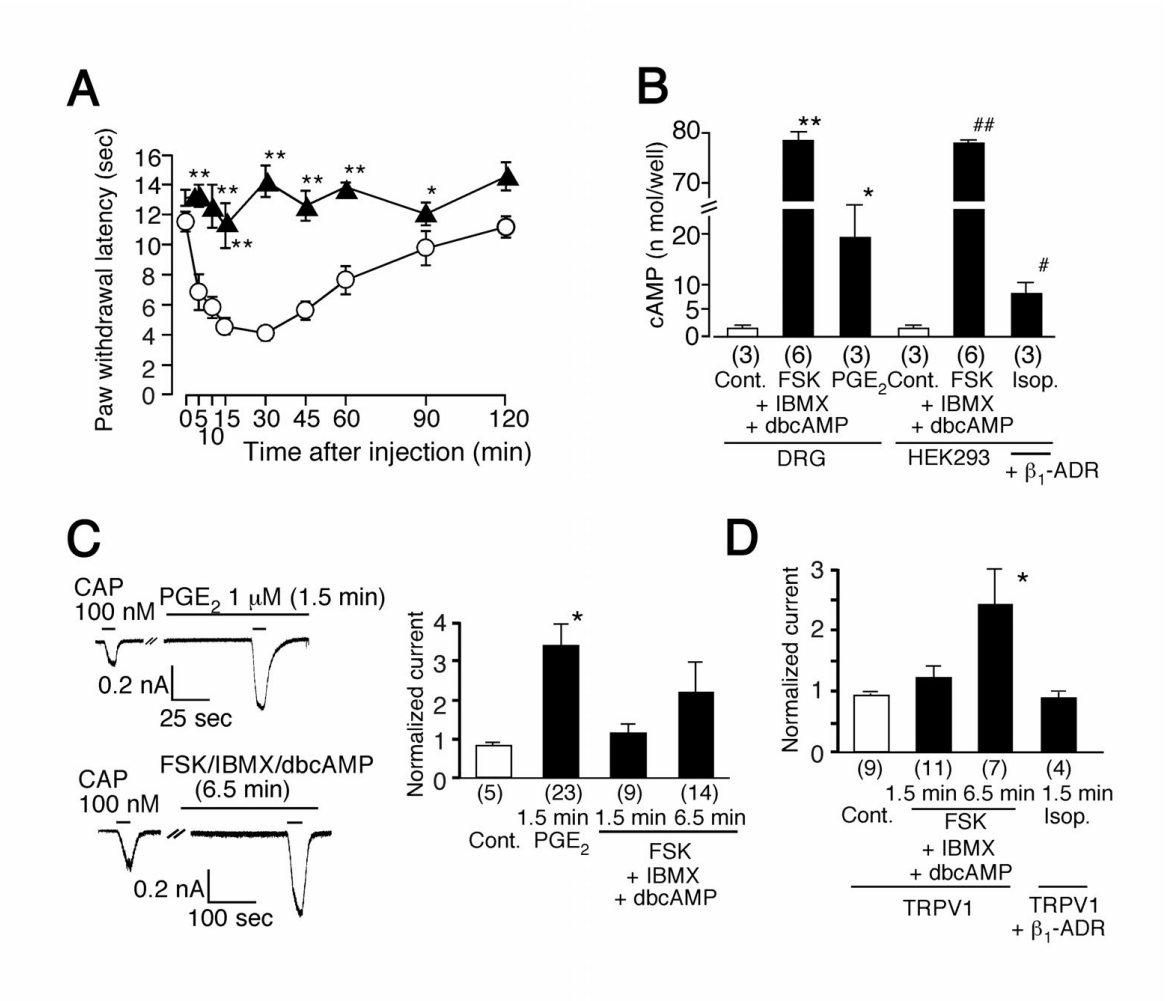
Physiological interaction of PGE_2 _with TRPV1 in mice. (**A**) PGE_2_-induced thermal hyperalgesia in TRPV1^+/+ ^mice (○, n = 6) or TRPV1^-/- ^mice (▲, n = 6). Reduction of paw withdrawal latency (thermal hyperalgesia) by intraplantar PGE_2 _(500 pmol/ 20 μL) injection was significantly diminished in TRPV1^-/- ^mice. * p < 0.05, ** p < 0.01 vs. TRPV1^+/+ ^mice. (**B**) Intracellular cAMP levels in mouse DRG neurons or HEK293 cells treated with a mixture of forskolin (FSK, 10 μM), IBMX (1 mM) and dibutyryl cAMP (dbcAMP, 3 mM), or PGE_2 _(1 μM) or isoproterenol (Isop., 10 μM). *, # p < 0.05 vs. Cont., **, ## p < 0.01 vs. Cont. (**C**) Representative traces of potentiation of capsaicin (100 nM)-activated current by extracellular PGE_2 _(1 μM, 1.5 min) or a mixture of FSK(10 μM), IBMX (1 mM) and dbcAMP (3 mM) (6.5 min) in mouse DRG neurons. Currents were normalized to values induced by first capsaicin application in the absence of additives (bar graph). Capsaicin was reapplied 1.5 or 6.5 min after exposure to bath solution with additives. Numbers in parenthesis indicate cells tested. * p < 0.05 vs. Cont. Holding potential (V_h_): -60 mV. (**D**) Long (6.5 min) but not short (1.5 min) activation of PKA pathway has effect on TRPV1 responses in HEK293 cells. FSK (10 μM), IBMX (1 mM) and dbcAMP (3 mM) were applied to cells expressing rat TRPV1. Isop. (10 μM) was applied to cells expressing both rat TRPV1 and β_1_-adrenergic receptors (β_1_-ADR). Numbers in parenthesis indicate cells tested. V_h_: -60 mV. * p < 0.05 vs Cont.

In order to examine the possibility that TRPV1 is involved in PGE_2_-induced hyperalgesia *in vivo*, we performed a behavioral analysis using wild type and TRPV1-deficient (TRPV1^-/-^) mice. PGE_2 _(500 pmol/20 μL) produced a significant reduction in paw withdrawal latency in response to radiant heat (thermal hyperalgesia) at 5 to 90 min following intraplantar injection in wild type mice (Figure 1A). On the other hand, the PGE_2_-induced thermal hyperalgesia was almost completely abolished in TRPV1^-/- ^mice, suggesting a functional interaction between PGE_2 _and TRPV1 (Figure 1A), consistent with a previous report that capsaicin-ablation of primary afferent neurons prevents PGE_2_-induced thermal hyperalgesia [23]. We next examined the interaction between PGE_2 _and TRPV1 in mouse DRG neurons using the patch-clamp technique. Capsaicin (100 nM) evoked small inward currents in DRG neurons. The capsaicin-evoked currents were significantly potentiated by 1.5 min pretreatment with PGE_2 _(1μM) in 19 of 23 cells as previously reported [21] (Figure 1C) (3.36 ± 0.55 fold increase, n = 23 for PGE_2 _(+); 0.78 ± 0.08 fold for PGE_2 _(-) (Cont.), n = 5, p < 0.05). Because it has been suggested that a PKA-dependent pathway is predominantly involved in the PGE_2_-induced potentiation of capsaicin-activated currents in rat DRG neurons [21], we examined the potential involvement of such a mechanism both in mouse DRG neurons and human embryonic kidney-derived HEK293 cells expressing TRPV1. No potentiation of the capsaicin-activated current responses was observed in DRG neurons treated with a mixture of forskolin (FSK, 10 μM), 3-isobutyl-1-methylxanthine (IBMX, 1 mM) and dibutyryl-cAMP (dbcAMP, 3 mM) for the same time period (1.5 min) (1.15 ± 0.20 fold increase, n = 9) although a significant increase in cAMP level was confirmed during such the treatment (Figures 1B and 1C). When we treated cells longer (6.5 min), 7 out of 14 cells showed increase of capsaicin-activated currents (2.15 ± 0.77 fold increase, n = 14, p = 0.28) (Figure 1C). In HEK293 cells, two different short (1.5 min) treatments to activate PKA produced no potentiation (Figure 1D) (treatment with a mixture of FSK, IBMX and dbcAMP in cells expressing TRPV1, 1.20 ± 0.19 fold increase, n = 11, and treatment with isoproterenol (Isop.) in cells expressing both TRPV1 and mouse β_1_-adrenergic receptors (β_1_-ADR), 0.83 ± 0.12 fold increase, n = 4) although a significant increase in cAMP level was confirmed following both treatments in HEK293 cells (Figure 1B). We also examined the effects of long treatment (6.5 min) with a mixture of FSK, IBMX and dbcAMP. This treatment caused significant potentiation of capsaicin-activated currents (2.39 ± 0.60 fold increase, n = 7, p < 0.05) (Figure 1D). These results suggest that both PKA-dependent and -independent pathways are involved in the potentiation of the capsaicin-activated currents by PGE_2_, that it takes longer to cause potentiation of capsaicin-activated currents through a PKA-dependent pathway, and that the PKA-independent pathway is predominantly involved under the short treatment conditions. Indeed, it has been reported that capsaicin-activated currents were not increased upon FSK/IBMX or 8-bromo-cAMP (8-Br-cAMP)/IBMX treatment in *Xenopus *oocytes expressing TRPV1, or treatment with isoproterenol in oocytes expressing both TRPV1 and β_1_-ADR [24].

### PGE_2 _increases TRPV1 activity through EP_1 _receptors

**Figure 2:**
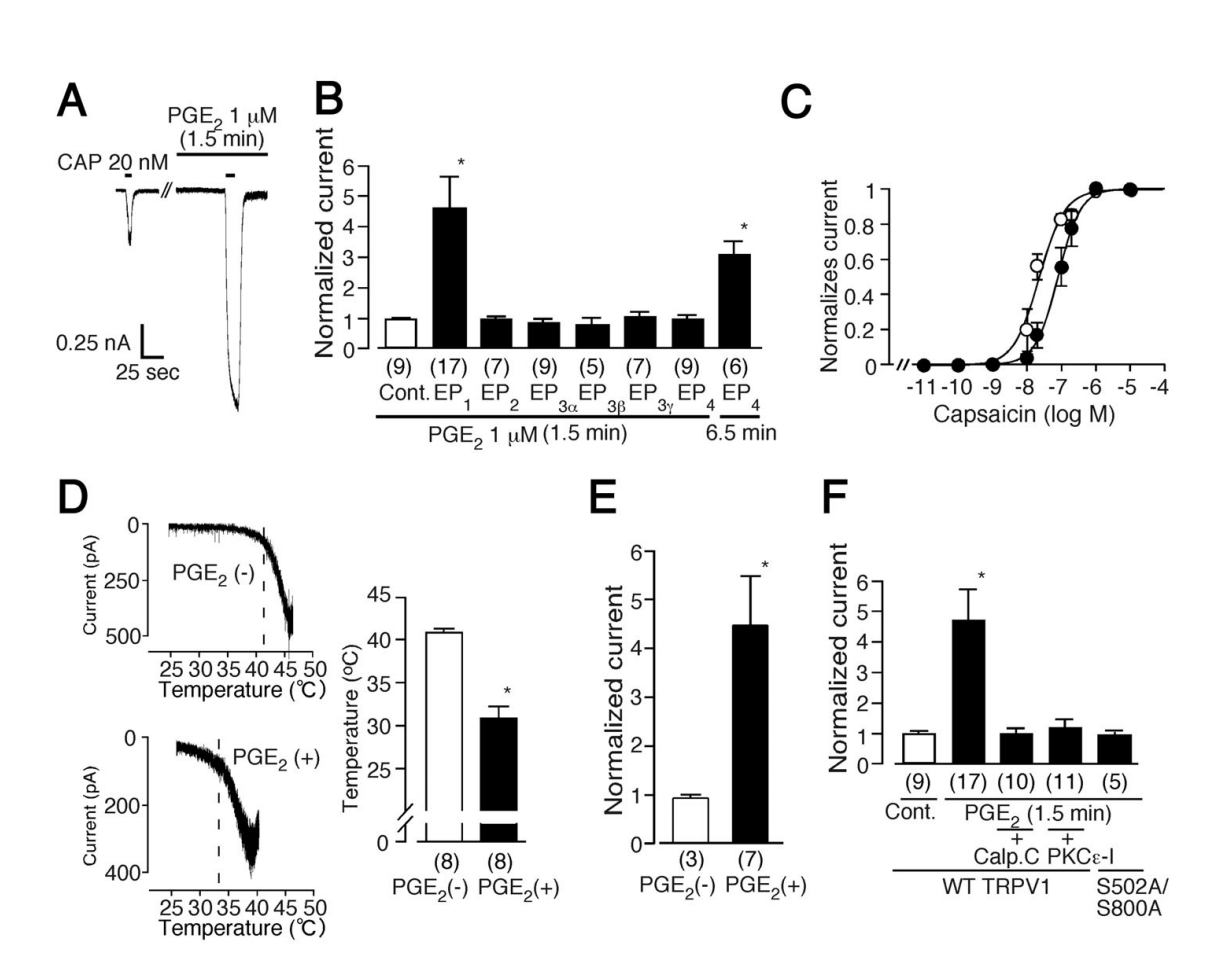
PGE_2 _increases TRPV1 activity through EP_1 _receptors in a PKC-dependent manner in HEK293 cells. (**A**) and (**B**) Treatment with PGE_2 _(1.5 min) potentiates capsaicin-evoked responses in cells expressing rat TRPV1 with mouse EP_1 _receptors, but not with other mouse EP receptors. Cells were pretreated with PGE_2 _(1 μM) for 1.5 or 6.5 min before second capsaicin (20 nM) application. V_h_: -60 mV. Currents were normalized as described in Figure 1. * p < 0.05 vs. control (Cont.). Numbers in parenthesis indicate cells tested. (**C**) Capsaicin dose-response curves for TRPV1 activation in the absence (•) and presence (○) of extracellular 1 μM PGE_2_. Currents were normalized to the current maximally activated by 1 μM capsaicin in the absence of PGE_2_. Figure shows averaged data fitted with the Hill equation. EC_50 _= 81.0 nM and Hill coefficient = 1.33 in the absence of PGE_2_. EC_50 _= 27.6 nM and Hill coefficient = 1.01 in the presence of PGE_2_. Data were obtained from 54 different cells. (**D**) Temperature threshold for TRPV1 activation was reduced in the presence of extracellular PGE_2 _(1 μM). Representative temperature-response profiles in the absence (upper) and presence (lower) of PGE_2 _(left). Temperature threshold for TRPV1 activation in the presence of PGE_2 _(30.6 ± 1.1°C) was significantly lower than that in the absence of PGE_2 _(40.7 ± 0.3°C) (right). * p < 0.05 vs. PGE_2 _(-). Numbers in parenthesis indicate cells tested. (**E**) Proton-evoked TRPV1 responses were significantly potentiated by PGE_2 _(1 μM). * p < 0.01 vs. PGE_2 _(-). (**F**) PKC-dependent pathway is involved in the PGE_2 _(1 μM, 1.5 min)-induced potentiation of capsaicin-activated currents. In some experiments, calphostin C (Calp. C) (1 μM) or PKCε translocation inhibitor (PKCε-I) (200 μM) was included in the pipette solution. Currents were normalized as described in Figure 1. Numbers in parenthesis indicate cells tested. * p < 0.05 vs. Cont. V_h_: -60 mV.

To explore the mechanism underlying the PKA-independent PGE_2 _(1.5 min)-induced potentiation of the capsaicin-evoked responses observed in DRG neurons, we first examine the effects of PGE_2 _on capsaicin-activated currents in HEK293 cells expressing TRPV1 and each EP receptor. PGE_2 _(1 μM, 1.5 min) caused a robust increase in the magnitude of low dose (20 nM) capsaicin-activated currents in HEK293 cells co-expressing TRPV1 with EP_1 _(0.90 ± 0.04 fold increase, n = 9 for control (Cont.); 4.60 ± 1.03 fold, n = 17 for PGE_2_, p < 0.05) (Figures 2A and 2B). This increase lasted more than three minutes, as we previously reported for PAR-2 (proteinase activated receptor 2)-mediated potentiation of TRPV1 activity [16]. In contrast, no such potentiation was detected in cells expressing TRPV1 with other EP receptor subtypes (0.91 ± 0.09 fold increase, n = 7; 0.77 ± 0.13, n = 9; 0.72 ± 0.24, n = 5; 0.98 ± 0.18, n = 7; 0.89 ± 0.15, n = 9 for EP_2_, EP_3α_, EP_3β_, EP_3γ _or EP_4_, respectively) (Figure 2B). Protracted (6.5 min) treatment with PGE_2 _caused a significant increase in capsaicin-activated currents in cells expressing TRPV1 and EP_4_, a phenomenon like that observed following treatment with a mixture of FSK, IBMX and dbcAMP (3.03 ± 0.48 fold increase, n = 6, p < 0.05 vs. Cont.) (Figure 2B), suggesting that the EP_4 _receptor, known to be expressed in DRG and coupled to Gs protein, is the receptor that activates a PKA-dependent signaling pathway upon prostaglandin exposure. All cells exhibiting an increase of capsaicin-activated currents upon treatment with a mixture of FSK, IBMX and dbcAMP also showed an increase in current in the presence of PMA (data not shown), suggesting that both PKA- and PKC-dependent pathways work in the same cells. To examine how PGE_2 _changes TRPV1 responsiveness, we measured TRPV1 current in single cells by applying a range of concentrations of capsaicin in the absence or presence of PGE_2_. The currents were normalized to the maximal current produced by 1 μM capsaicin in each cell. Maximal current in the presence of PGE_2 _was almost the same as that in the absence of PGE_2_. The resultant dose-response curves clearly demonstrate that PGE_2 _enhances capsaicin action on TRPV1 by lowering EC_50 _values without altering maximal responses (EC_50 _from 81.0 nM to 27.6 nM) (Figure 2C). We next examined the effects of PGE_2 _on the thermal sensitivity of TRPV1. When temperature ramps were applied to HEK293 cells expressing both TRPV1 and EP_1 _in the absence of PGE_2_, heat-evoked currents developed at 40.7 ± 0.3°C (n = 8) (Figure 2D). In contrast, the temperature threshold for TRPV1 activation was significantly reduced to 30.6 ± 1.1°C in the presence of PGE_2 _(n = 8, p < 0.05) (Figure 2D) implying that under these conditions, TRPV1 could be activated at normal body temperature. A similar potentiating effect of PGE_2 _was observed for proton (pH 6.2)-evoked TRPV1 current responses (0.91 ± 0.06 fold increase, n = 3 for control; 4.47 ± 1.09 fold, n = 7 for PGE_2_, p < 0.01) (Figure 2E). These data clearly show that TRPV1 currents evoked by any of three different stimuli (capsaicin, proton, or heat) are potentiated or sensitized by PGE_2 _through EP_1 _receptor activation. On the other hand, the temperature threshold for TRPV1 activation was not changed upon treatment with a mixture of FSK, IBMX and dbcAMP in HEK293 cells expressing TRPV1 (40.8 ± 0.8°C, n = 4), suggesting different actions on TRPV1 by PKA and PKC.

The signaling pathway downstream of EP_1 _remains to be clarified. We have reported that G_q/11_-coupled metabotropic receptor activation such as ATP (P2Y), bradykinin (B2) and proteinase-activated receptor 2 (PAR2) receptors causes potentiation or sensitization of TRPV1 through the PKC-dependent phosphorylation of TRPV1 [16-18,25]. Therefore, we examined whether a similar signal transduction pathway is involved in the regulation of TRPV1 responses through EP_1_. When calphostin C (Calp.C), a specific PKC inhibitor, was added to the pipette solution, the effect of PGE_2 _was almost completely inhibited (0.92 ± 0.15 fold increase, n = 10) (Figure 2F). Similarly, a PKCε translocation inhibitor (PKCε-I) abolished the potentiation of TRPV1 response by PGE_2 _(1.11 ± 0.25 fold increase, n = 11) (Figure 2F). These data suggest that PGE_2_-induced potentiation of TRPV1 responsiveness develops through activation of PKCε. To further confirm the involvement of PKC-dependent phosphorylation, PGE_2 _effects were examined using cells expressing a TRPV1 mutant, S502A/S800A which is insensitive to PKC-dependent phosphorylation [19]. No potentiation of capsaicin-activated currents was observed upon PGE_2_treatment of cells expressing S502A/S800A (0.85 ± 0.15 fold increase, n = 5) (Figure 2F), further indicating the involvement of PKC-dependent phosphorylation. Since S502 is a PKA-phosphorylation site as well [26], we examined the effects of treatment with a mixture of FSK, IBMX and dbcAMP on the capsaicin-activated currents in cells expressing S502A/S800A. Such treatment failed to potentiate the capsaicin-activated currents (1.13 ± 0.07 fold increase, n = 10), suggesting that S502 is a substrate for PKA-dependent phosphorylation of TRPV1 as well.

### Sensitization of TRPV1 by EP_1 _receptors in mouse

**Figure 3:**
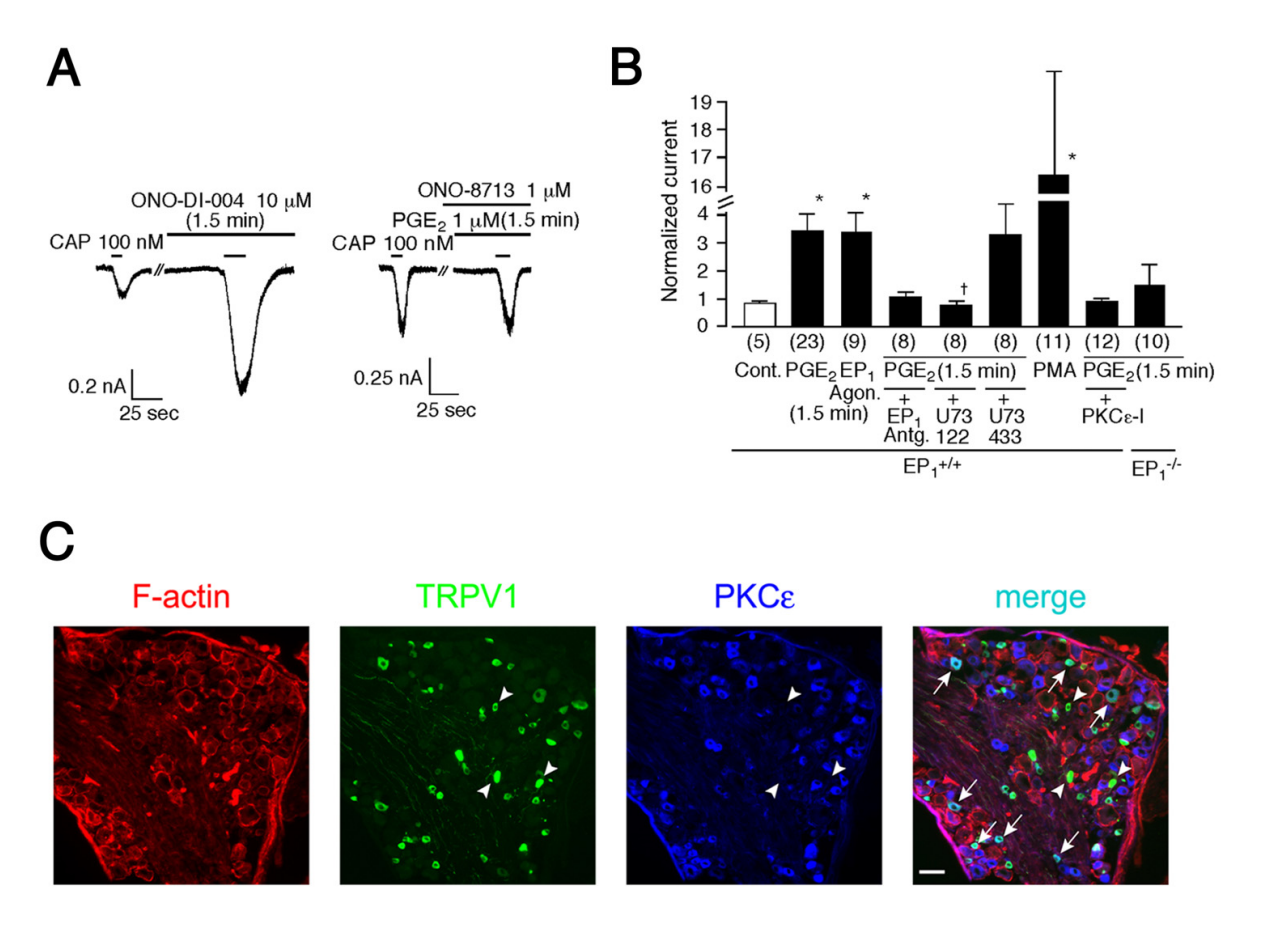
EP_1 _receptor involvement in PGE_2 _(1.5 min)-induced potentiation of capsaicin-activated currents in mouse DRG neurons. (**A**) Representative traces of potentiation of capsaicin-activated currents by a specific EP_1 _agonist, ONO-DI-004 (10 μM, 1.5 min), and reverse of the PGE_2 _(1.5 min)-induced potentiation by a specific EP_1 _antagonist, ONO-8713 (1 μM). V_h_: -60 mV. (**B**) Effects of PGE_2 _(1 μM), ONO-DI-004 (EP_1 _Agon., 10 μM), PGE_2 _plus ONO-8713 (EP_1 _Antg., 1 μM), PGE_2 _plus U73122 (3 μM), PGE_2 _plus U73343 (3 μM), phorbol 12-myristate 13-acetate (PMA, 100 nM) or PGE_2 _plus PKCε-I (200 μM) on capsaicin-activated currents in DRG neurons from wild type (EP_1_^+/+^) mice, and effects of PGE_2 _on capsaicin-activated currents in DRG neurons from EP_1_^-/- ^mice. Currents are normalized as described in Fig. 1. * p < 0.05 vs. Cont., + p < 0.05 vs. U73343. Numbers in parenthesis indicate cells tested. (**C**) Co-expression of TRPV1 (green) and PKCε (blue) in mouse DRG. Arrowheads indicate neurons positive for TRPV1 but not for PKCε. Arrows indicate neurons positive for both TRPV1 and PKCε (light blue). Bar, 100 μm.

To examine the involvement of EP_1 _in PGE_2 _(1.5 min)-induced potentiation of capsaicin-evoked response in native neurons, we used a specific EP_1 _agonist, ONO-DI-004 [27], and a specific EP_1 _antagonist, ONO-8713 [28], in mouse DRG neurons. ONO-DI-004 was found to significantly increase the capsaicin-activated currents to an extent similar to that observed with PGE_2 _(3.36 ± 0.68 fold increase for PGE_2_, n = 23, p < 0.05 vs. control (Cont.); 3.30 ± 0.68 fold for ONO-DI-004 (EP_1 _Agon.), n = 9, p < 0.05 vs. Cont.) (Figures 3A left and 3B). Furthermore, potentiation of the capsaicin-activated currents by PGE_2 _was inhibited in the presence of ONO-8713 (EP_1 _Antg., 1.00 ± 0.17 fold increase, n = 8) (Figures 3A right and 3B). These results indicate that PGE_2 _(1.5 min)-induced potentiation of the capsaicin-activated current responses occurs through EP_1 _receptors in DRG neurons. To confirm the involvement of PKC-dependent events downstream of PGE_2 _effects in DRG neurons, we first examined the effects of a specific phospholipase C (PLC) inhibitor, U73122 (3 μM). PGE_2_-induced potentiation of capsaicin-activated current was significantly diminished in the presence of U73122 while control U73343 exhibited no such effects (0.73 ± 0.11 fold increase, n = 8 for U73343; 3.40 ± 1.11 fold, n = 8 for U73433, p < 0.05) (Figure 3B). Furthermore, PGE_2 _failed to potentiate the capsaicin-activated currents when PKCε-I was included in the pipette solution (0.86 ± 0.09 fold increase, n = 12) (Figure 3B). A robust potentiating effect of phorbol 12-myristate 13-acetate (PMA, 100 nM) also supported the involvement of PKC-dependent events (16.36 ± 3.68 fold increase, n = 11, p < 0.05) (Figure 3B). To further confirm the involvement of EP_1 _receptors, DRG neurons of EP_1 _deficient mice (EP_1_^-/-^) were subjected to patch-clamp analysis. PGE_2 _failed to potentiate capsaicin-activated currents in the DRG neurons from EP_1_^-/- ^mice (1.45 ± 0.70 fold increase, n = 10) (Figure 3B). Functional interaction of PKCε with TRPV1 prompted us to examine the expression of the two proteins in mouse DRG. Three hundred seventy eight out of 541 TRPV1 positive neurons (69.9 %) were stained with anti-PKCε antibody, supporting the TRPV1 activation pathway through PKCε (Figure 3C).

**Figure 4:**
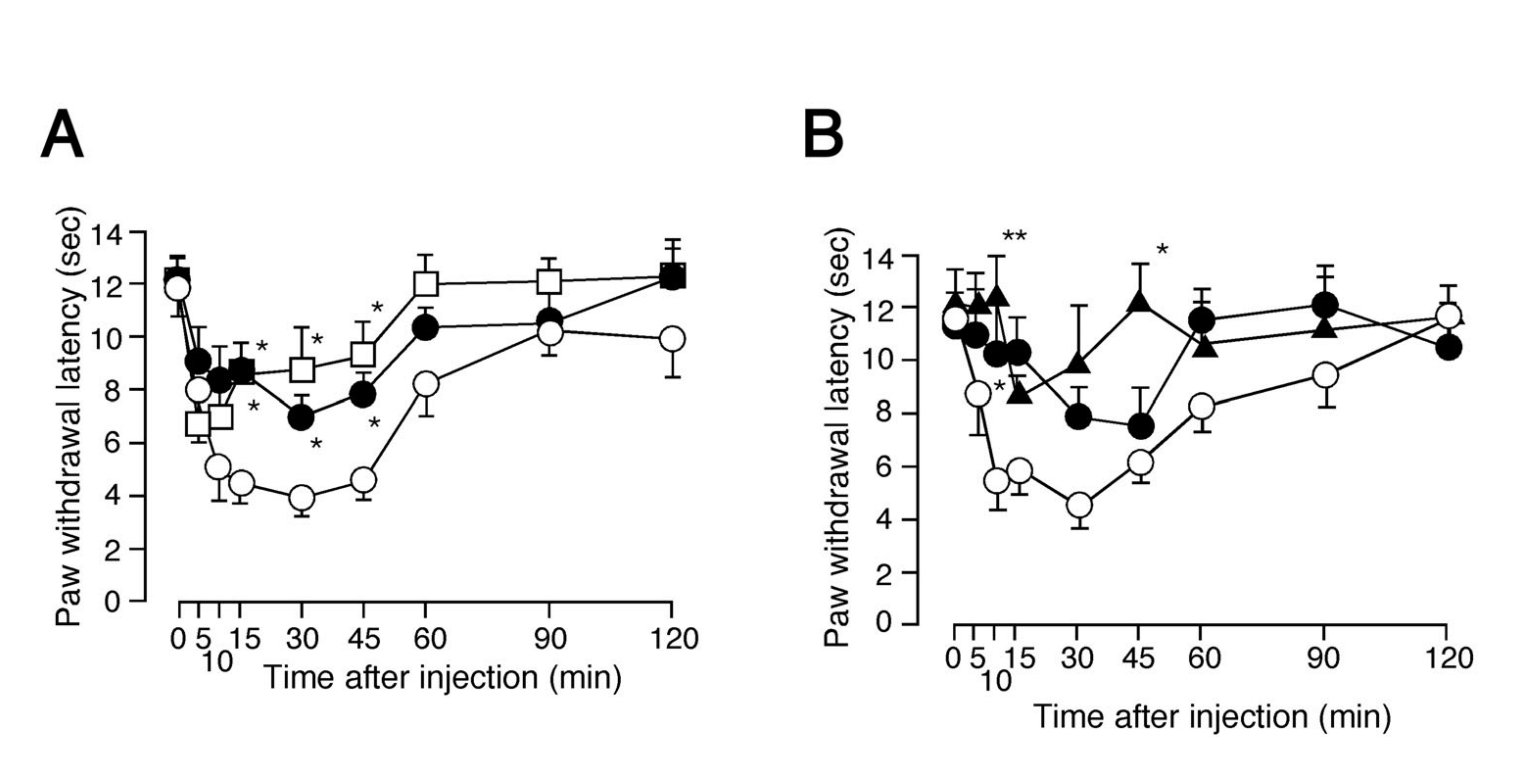
Interaction between TRPV1 and EP_1 _receptors in a behavioral level. (**A**) PGE_2_-induced thermal hyperalgesia in wild type mice with (▴, n = 6) or without (○, n = 6) pretreatment (ONO-8713, 500 pmol/ 20 μL), or in EP_1_^-/- ^mice (•, n = 6). * p < 0.05 vs. wild type mice. (**B) **10% Mustard oil-induced thermal hyperalgesia in wild type mice (○, n = 12), TRPV1^-/- ^mice (▲, n = 6) or EP_1_^-/- ^mice (•, n = 6). * p < 0.05, ** p < 0.01 vs. wild type mice.

We next investigated the involvement of EP_1 _receptors in PGE_2_-induced thermal hyperalgesia at the behavioral level. PGE_2_-induced thermal hyperalgesia was significantly diminished at 15 to 45 min after injection in EP_1_^-/- ^mice (Figure 4A), relative to that observed in wild type mice. The involvement of EP_1 _receptors in the PGE_2_-induced hypersensitivity was supported by another behavioral analysis in which PGE_2 _caused less reduction of paw withdrawal latency in wild type mice pretreated with a specific EP_1 _antagonist (500 pmol/ 20 μL) than in vehicle control (Figure 4A). These results suggest that a PKC-dependent pathway downstream of EP_1 _activation is mainly involved in PGE_2_-induced thermal hyperalgesia. We have hypothesized that the potentiation of TRPV1 activity by several inflammatory mediators could represent one important mechanism underlying acute inflammatory pain sensation. To prove the accuracy of this hypothesis, we investigated the involvement of EP_1 _in inflammatory pain-related responses using mustard oil which is known to cause inflammation [29,30]. Topical application of mustard oil induced clear thermal hyperalgesia (Figure 4B). The mustard oil-induced thermal hyperalgesia was significantly reduced both in TRPV1^-/- ^mice and EP_1_^-/- ^mice. Thus, these data show that EP_1 _contributes to inflammatory nociception in mice and support the hypothesis.

### Sensitization of TRPV1 by IP receptors

**Figure 5:**
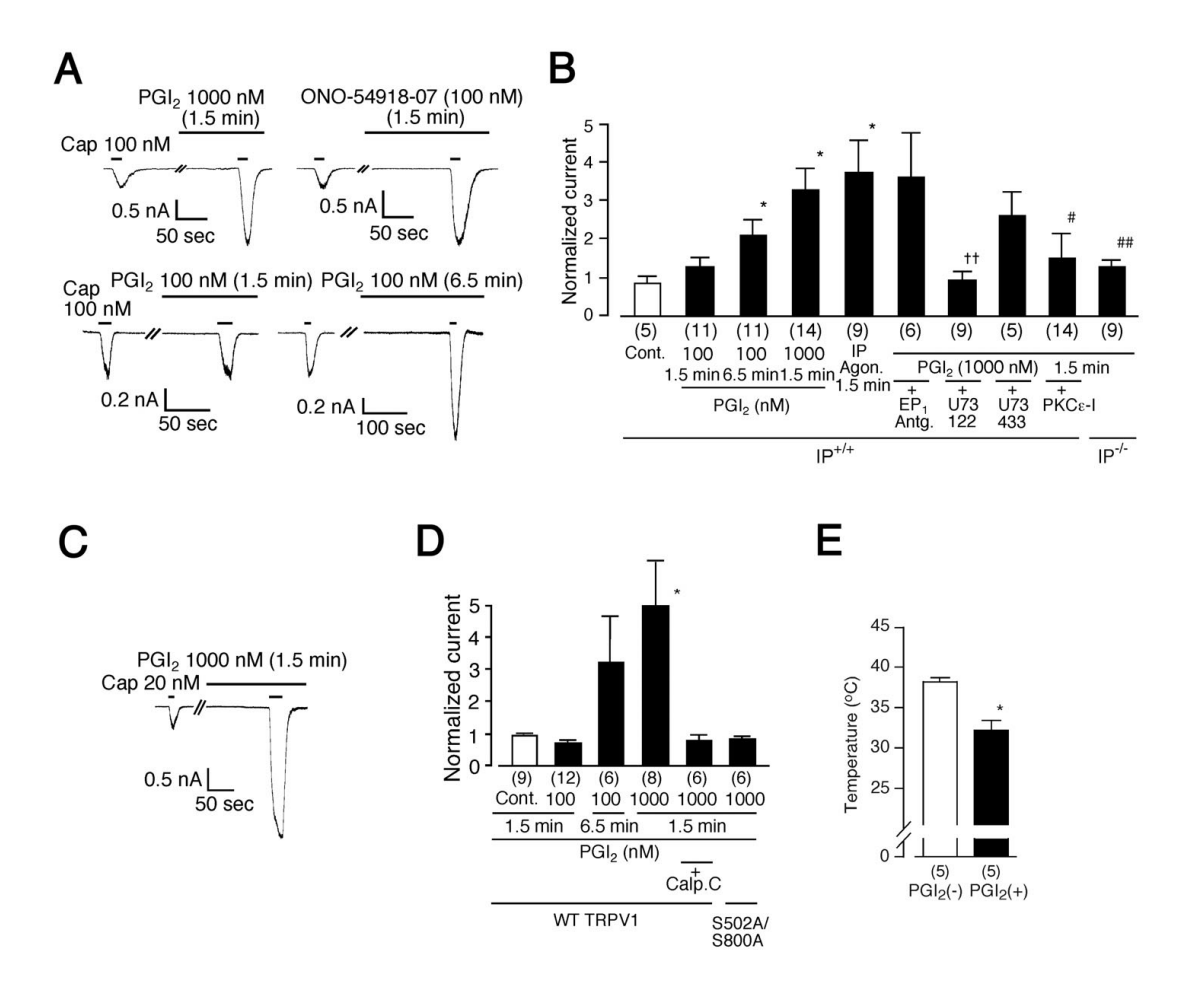
PGI_2 _causes potentiation or sensitization of TRPV1 through mainly through PKC activation. (**A**) Representative traces of potentiation of capsaicin-activated currents by PGI_2 _(1000 nM, 1.5 min), a specific IP agonist, ONO-54918-07 (1.5 min) or PGI_2 _(100 nM, 6.5 min), but not by PGI_2 _(100 nM, 1.5 min) in mouse DRG neurons. V_h_: -60 mV. (**B**) Effects of treatments (1.5 or 6.5 min) with PGI_2 _(100 or 1000 nM), ONO-54918-07 (IP Agon., 100 nM), PGI_2 _(1000 nM) plus ONO-8713 (EP_1 _Antg., 1 μM), PGI_2 _(1000 nM) plus U73122 (3 μM), PGI_2 _(1000 nM) plus U73343 (3 μM) or PGI_2 _(1000 nM) plus PKCε-I (200 μM) on capsaicin-activated currents in DRG neurons from wild type (IP^+/+^) mice, and effects of PGI_2 _on capsaicin-activated currents in DRG neurons from IP-deficient (IP^-/-^) mice. Currents are normalized as described in Figure 1. * p < 0.05 vs. Cont. ++ p < 0.01 vs. U73343, # p < 0.05, ## p < 0.01 vs. PGI_2 _(1000 nM, 1.5 min) in DRG neurons from IP^+/+ ^mice. Numbers in parenthesis indicate cells tested. (**C**) A representative trace of potentiation of capsaicin-activated currents by PGI_2 _(1000 nM, 1.5 min) in HEK293 cells expressing both TRPV1 and IP. V_h_: -60 mV. (**D**) Effects of treatments (1.5 or 6.5 min) with PGI_2 _(100 or 1000 nM) or PGI_2 _(1000 nM) plus calphostin C (Calp. C, 1 μM) on capsaicin-activated currents in HEK293 cells expressing rat wild type TRPV1 or S502A/S800A mutant with IP. Currents are normalized as described in Figure 1. * p < 0.05 vs. Cont. (**E**) Temperature threshold for TRPV1 activation in the presence of PGI_2 _(32.2 ± 1.2°C) was significantly lower than that in the absence of PGI_2 _(38.2 ± 0.5°C) in HEK293 cells expressing rat TRPV1 and IP. * p < 0.01 vs. PGI_2 _(-).

In order to determine whether the observed responses are specific to PGE_2_, we extended our analysis to PGI_2 _whose receptor has been reported to be involved in nociception [8]. We first examined the effects of PGI_2 _on capsaicin-activated currents in mouse DRG neurons. PGI_2 _pretreatment (1000 nM, 1.5 min) potentiated capsaicin (100 nM)-activated currents (3.23 ± 0.55 fold increase, n = 14 or 0.78 ± 0.08 fold, n = 5 with or without (Cont.) PGI_2_, respectively; p < 0.05) whereas at 100 nM, PGI_2 _(1.5 min) showed no such effects (1.24 ± 0.22 fold, n = 11) (Figures. 5A and 5B). On the other hand, long (6.5 min) treatment with PGI_2 _(100 nM) caused significant potentiation of capsaicin-activated currents as in the treatment with a mixture of FSK, IBMX and dbcAMP (2.06 ± 0.54 fold increase, n = 11, p < 0.05 vs. Cont.) (Figures 5A and 5B). The potentiation effects of PGI_2 _appear to occur through IP receptors because a specific IP agonist, ONO-54918-07 (100 nM) [31] caused similar potentiation of capsaicin-activated currents (Agon., 3.71 ± 0.81 fold increase, n = 9, p < 0.05 vs. Cont.) (Figures 5A and 5B) although PGI_2 _is known to cross react with some EP receptors [2]. The fact that a specific EP_1 _antagonist, ONO-8713 failed to prevent the PGI_2_-induced potentiation (+EP_1 _Antg., 3.55 ± 1.17 fold increase, n = 6) (Figure 5B) further suggests the involvement of IP receptors in the potentiation process. The involvement of IP receptors in the PGI_2_-induced potentiation of capsaicin-activated currents was further supported by the ineffectiveness of PGI_2 _on DRG neurons from IP-deficient mice (IP^-/-^) (1.25 ± 0.16 fold increase, n = 9, p < 0.01 vs. 1000 nM of PGI_2_) (Figure 5B). It has been reported that low concentrations of PGI_2 _stimulate Gs protein coupled to IP receptors whereas high concentrations of PGI_2 _stimulate not only Gs but also Gq [32]. This property might explain the dose-dependent effects of PGI_2 _on capsaicin-activated currents: PKC-dependent sensitization of TRPV1 occurs downstream of Gq-coupled IP receptor activation at high concentrations (1000 nM) of PGI_2 _(1.5 min) while long (6.5 min) treatment with low concentrations (100 nM) of PGI_2 _causes potentiation of TRPV1 activity through Gs activation. To test this hypothesis, PGI_2 _(1.5 min)-induced potentiation of capsaicin-activated currents was examined in the presence of U73122. When U73122 was included in the pipette solution, PGI_2 _(1.5 min) failed to potentiate the currents whereas U73343 exhibited no such effects, indicating the involvement of PLC activation in the potentiating process (0.97 ± 0.40 fold increase, n = 9 for U73122, 2.58 ± 0.66 fold, n = 5 for U73343, p < 0.05) (Figure 5B). Furthermore, PKCε-I included in the pipette solution almost completely blocked the PGI_2 _(1000 nM)-induced potentiation (1.49 ± 0.60 fold increase, n = 14), suggesting the involvement of PKCε-dependent regulation mechanism (Figure 5B).

**Figure 6:**
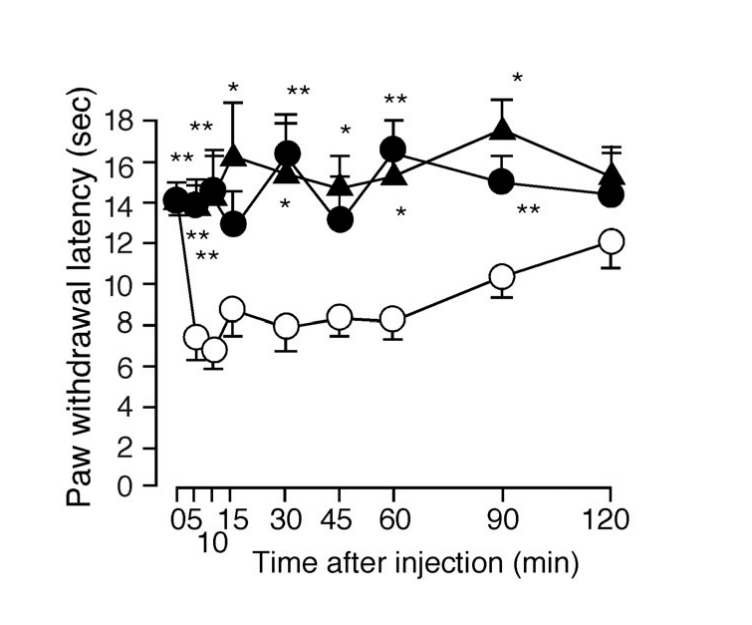
Interaction between TRPV1 and IP receptors at a behavioral level. PGI_2_-induced thermal hyperalgesia in wild type mice (○, n = 6), TRPV1^-/- ^mice (▲, n = 6) or IP^-/- ^mice (•, n = 6). Thermal hyperalgesia by intraplantar PGI_2 _(500 pmol/ 20 μL) injection was significantly diminished in TRPV1^-/- ^mice and IP^-/- ^mice. * p < 0.05, ** p < 0.01 vs. wild type mice.

Dose-dependent PGI_2 _(1.5 min)-induced potentiation of capsaicin-activated currents was also observed in HEK293 cells expressing TRPV1 and IP receptors (0.90 ± 0.04 fold increase, n = 9 without PGI_2 _(Cont.); 0.68 ± 0.08 fold, n = 12 with 100 nM of PGI_2_; 0.75 ± 0.07 fold, n = 6 with 300 nM PGI_2_, 4.96 ± 1.36 fold, n = 8 with 1000 nM of PGI_2_, p < 0.01 vs. Cont.) (Figures 5C and 5D, and data not shown). Calp. C blocked PGI_2_-induced potentiation of TRPV1 currents (0.75 ± 0.15 fold increase, n = 6) (Figure 5D). Furthermore, PGI_2 _(1000 nM) failed to potentiate capsaicin-activated currents in HEK293 cells expressing the S502/S800 mutant (0.80 ± 0.05 fold, n = 6) (Figure 5D). Long (6.5 min) treatment with PGI_2 _(100 nM) caused an increase in capsaicin-activated currents in 4 out of 6 cells, as did long treatment with a mixture of FSK, IBMX and dbcAMP in HEK293 cells expressing TRPV1 (3.19 ± 1.45 fold increase, n = 6, p = 0.16). These results suggest that a mechanism involving PKC is predominantly involved in the regulation of TRPV1 activity during short treatment with PGI_2 _although both PKA-dependent and PKC-dependent pathways may contribute. The temperature threshold for TRPV1 activation was significantly reduced (from 38.2 ± 0.5°C, n = 5 to 32.2 ± 1.2°C, n = 5) in the presence of PGI_2_, suggesting the possibility that IP receptor activation can cause nociception at body temperature (Figure 5E). Finally, PGI_2_-induced thermal hyperalgesia observed in wild type mice disappeared almost completely in both TRPV1-deficient (TRPV1^-/-^) mice and IP-deficient (IP^-/-^) mice, suggesting that the functional interaction of TRPV1 with IP causes thermal hyperalgesia at the behavioral level (Figure 6).

## Discussion

The data presented herein demonstrate that TRPV1 is essential for the development of thermal hyperalgesia *in vivo *induced by two major inflammation-associated prostaglandins, PGE_2 _and PGI_2_, and that TRPV1 and EP_1 _or IP receptors can functionally interact, mainly through a PKC-dependent pathway. The temperature threshold for TRPV1 activation is reduced below 35°C in the presence of prostaglandins, so that TRPV1 can be activated at normal body temperature, possibly leading to spontaneous pain sensation. This interaction might be one important underlying mechanism for the well-recognized peripheral nociceptive actions of PGE_2 _or PGI_2 _in the context of inflammation. In the present study, 1 μM PGE_2 _or PGI_2 _was found to potentiate or sensitize TRPV1 activity. It is not well known how much PGE_2 _or PGI_2 _is released locally at the site of inflammation. However, more than micromolar-order concentrations of PGE_2 _and PGI_2 _have been reported to be synthesized by macrophages upon lipopolysacharide (LPS) stimulation [33,34], suggesting that 1 μM is an attainable concentration in the context of inflammation. It has been previously reported that EP_1 _is coupled to intracellular Ca^2+ ^mobilization in CHO cells [35]. However, the transduction events downstream of EP_1 _signaling have been unclear. Together with a report suggesting the possible coupling of EP_1 _with G_q/11_-protein [36], our data indicate that EP_1 _receptors activate a PKC-dependent signal transduction pathway.

There has been extensive work demonstrating the activation of a PKA-dependent pathway by PGE_2 _that influences capsaicin- or heat-mediated actions in rat sensory neurons [20-22,37,38] as well as interactions between cloned TRPV1 and PKA [26,39-42]. These results suggest that PKA plays a pivotal role in the development of hyperalgesia and inflammation by prostaglandins. In our experiments using mouse DRG neurons and HEK293 cells expressing TRPV1, a PKC-dependent pathway was found to be predominantly involved in both PGE_2 _(1.5 min)- and PGI_2 _(1.5 min)-induced responses. The reason that there has been no study describing the involvement of a PKC-dependent pathway in the regulation of TRPV1 following prostaglandin receptor activation is not clear. In the present study, it was found that both PKA- and PKC-dependent pathways are involved downstream of prostaglandin actions on TRPV1 although the PKC-dependent one appears to predominate. A PKA-dependent pathway took a relatively long time to exert its potentiating effects on TRPV1 activity, suggesting some difference between PKA- and PKC-dependent phosphorylation of TRPV1. Indeed, Bhave et al. treated cells with 8-Br-cAMP for 30 min to inhibit TRPV1 desensitization through phosphorylation [39], and significant potentiation of capsaicin-activated currents in rat DRG neurons was observed upon prolonged (greater than 10 min) exposure to PGE_2 _[21]. Furthermore, there is a report describing the ineffectiveness of PKA stimulation on TRPV1 currents in *Xenopus *oocytes treated with 8-Br-cAMP and IBMX for relatively short periods [24]. Both PKA-dependent and PKC-dependent pathways might work in concert in native cells. Patch-clamp recordings in the previous studies were performed in the Ca^2+^-containing solutions, whereas we did all of our experiments under Ca^2+^-free conditions, to avoid Ca^2+^-dependent TRPV1 desensitization [43]. Potentiation of capsaicin-activated currents by PGE_2 _was observed in embryonic rat DRG neurons [21] while we used adult mouse DRG neurons. Furthermore, potentiation of heat-activated currents [26], inhibition of desensitization of capsaicin-activated currents [39,41,44] or anandamide-induced cytosolic Ca^2+ ^increase [40] but not potentiation of capsaicin-activated current response were examined in the previous studies investigating the involvement of PKA-dependent pathway in TRPV1 activity. Thus, difference in experimental conditions or readout might also account for the different outcomes. The physiological relevance of the two different pathways downstream of prostaglandin exposure remains to be elucidated. The fact that only PKC activation leads to the reduction of temperature threshold for TRPV1 activation might be pertinent to this issue. Disruption of interaction between phosphatidylinositol-4, 5-bisphosphate (PIP_2_) and TRPV1 has also been reported to be involved in the sensitization of TRPV1 downstream of PLC activation [45,46]. In our study, however, both PGE_2_- and PGI_2_-induced potentiation of TRPV1 activity was completely inhibited by treatments with two kinds of PKC inhibitors. Thus, we believe that a PKC-dependent pathway is predominantly involved in the PGE_2_- and PGI_2_-induced potentiation or sensitization of TRPV1 activity in mice.

The inhibition of PGE_2_-induced thermal hyperalgesia observed in EP_1_^-/- ^mice, while significant, was not very robust, compared with that in TRPV1^-/- ^mice (Figure 4). Other pathways, most likely including one involving PKA, might account for the residual component. Further, inhibition of mustard oil-induced thermal hyperalgesia observed in TRPV1^-/- ^or EP_1_^-/- ^mice might seem not to be robust or dramatic (Figure 4). Since many inflammatory factors activating PLC-coupled receptors are involved in the inflammatory response [47,48]. In such a complicated environment, thermal hyperalgesia was significantly diminished in TRPV1^-/- ^mice or EP_1_^-/- ^mice albeit at a few time points, suggesting the importance of the two molecules in the context of inflammatory pain sensation. Given the fact that one of the final targets of both PGE_2 _and PGI_2 _is TRPV1 as shown in our study, compounds acting on EP_1_, IP or TRPV1, or interfering with their interaction could prove useful in the treatment of pain and inflammation.

## Conclusions

Potentiation or sensitization of TRPV1 activity through EP_1 _or IP activation, mainly through PKC- and PKA-dependent mechanisms, might be important mechanism underlying the peripheral nociceptive actions of PGE_2 _or PGI_2_.

## Methods

### Animals

Male C57BL/6-strain mice (4 weeks, SLC, Shizuoka, Japan), EP_1_-deficient mice (4 weeks, from Dr. Narumiya), IP-deficient mice (4 weeks, from Dr. Narumiya) or TRPV1-deficient mice (4 weeks, from Dr. Julius, UCSF) were used. They were housed in a controlled environment (12 h light/dark cycle, room temperature 22–24°C, 50–60% relative humidity) with free access to food and water. All procedures involving the care and use of mice were carried out in accordance with institutional (Mie University) guidelines and the National Institute of Health guide for the care and use of laboratory animals.

### Behavioral study

Thermal nociceptive threshold was assessed using the paw withdrawal test. Mice were placed in a transparent Perspex box on a thin glass platform (Plantar test, Ugo Basile, Italy). They were injected intraplantarly with PGE_2 _(500 pmol/ 20 μL, Sigma) with or without ONO-8713 (500 pmol/ 20 μL), or with PGI_2 _(500 pmol/ 20 μL, Sigma), or applied topically to the plantar surface of right hind paw with 10% mustard oil (Sigma) (diluted with mineral oil), and the paw withdrawal latency to radiant heat applied to the plantar surface of hind paw was measured as the time from onset of the radiant heat to the withdrawal of the mouse hind paw.

### Cell culture

Human embryonic kidney-derived (HEK293) cells were maintained in Dulbecco's modified Eagle's medium (Invitrogen; supplemented with 10% fetal bovine serum, penicillin, streptomycin and L-glutamine) and transfected with 0.5 μg of rat TRPV1 cDNA and 0.5 μg of mouse EP or IP receptor cDNAs (EP_1_, EP_2_, EP_3α_, EP_3β_, EP_3γ_, EP_4 _or IP) using Lipofectamine Plus Reagent (Invitrogen). Primary cultures prepared from adult C57BL/6-strain mice, EP_1_-deficient mice or IP-deficient mice dorsal root ganglion (DRG) neurons were incubated in medium containing nerve growth factor (Sigma, 100 ng/ml).

### Electrophysiology

Whole-cell patch-clamp recordings were performed 1 day after transfection to HEK293 cells or dissociation of the DRG neurons. Standard bath solution contained 140 mM NaCl, 5 mM KCl, 2 mM MgCl_2_, 5 mM EGTA, 10 mM HEPES, 10 mM glucose, pH7.4 (adjusted with NaOH). Pipette solution contained 140 mM KCl, 5 mM EGTA, 10 mM HEPES, pH7.4 (adjusted with KOH). All patch-clamp experiments were performed at room temperature (22°C). Thermal stimulation was applied by increasing the bath temperature at a rate of 1.0°C/sec with a preheated solution. When the heat-activated currents started to inactivate, the preheated solution was changed to a 22°C one. Chamber temperature was monitored with a thermocouple placed within 100 μm of the patch-clamped cell. For this analysis, heat-evoked current responses were compared between different cells, rather than within the same cell, because repetitive heat-evoked currents show significant desensitization even in the absence of extracellular Ca^2+ ^[13] and because the thermal sensitivity of TRPV1 increases with repeated heat application [49]. Threshold temperature for activation was defined as the intersection where two lines approximating the stable baseline current and the clearly increasing temperature-dependent current cross in the temperature-response profile. The sensitivity of DRG neurons to capsaicin is slightly lower than that of TRPV1-transfected HEK293 cells as previously reported [18,50]. Therefore, we applied capsaicin at 100 nM to DRG neurons and at 20 nM to HEK293 cells.

### cAMP measurement

Intracellular cAMP level was examined using 'cAMP Biotrak Enzymeimmunoassay System' according to the manufacture's direction (Amersham Biosciences). In brief, intracellular cAMP released upon membrane hydrolysis of treated cells (10,000 cells/ well) after stimulation (90 sec) was measured based on competition between unlabelled cAMP and a fix quantity of Peroxidase-labeled cAMP for a limited number of the binding sites on a cAMP specific antibody.

### Immunostaining

DRG was removed from male C57BL/6-strain mice and frozen in liquid nitrogen, and the frozen tissue was cut on a cryostat at a 10 μm thickness. The sections were incubated with the rabbit anti-rat TRPV1 polyclonal antibody (1: 500; Oncogene) and anti-rat PKCε monoclonal antibody (1: 250; Transduction lab) at 4°C for 2 days. Slides with the section were washed with PBS, followed by incubation with Alexa 488-conjugated goat anti-rabbit IgG (1: 700, Molecular Probes), Alexa 350-conjugated anti-mouse IgG (1: 500, Molecular Probes) and Texas Red-phalloidin (1: 500, Molecular Probes). Images were obtained using an Olympus fluorescent microscope with a cooled-CCD camera (ORCA-ER, Hamamatsu Photonics) and IP-Lab Image software (Scanalytics Inc.).

### Chemicals

ONO-DI-004, ONO-8713 and ONO-54918-07 were obtained from Ono Pharmaceutical Co., Ltd (Osaka, Japan). Calphostin C, phorbol 12-myristate 13-acetate, forskolin, 3-isobutyl-1-methylxanthine, dibutyryl-cAMP, isoproterenol, U73122 and U73343 were from Sigma, and PKCε translocation inhibitor was from Calbiochem.

### Statistics

Values are shown as the mean ± S.E. and data are analyzed using an unpaired *t *test. P values of < 0.05 were considered significant.

## Competing interests

The author(s) declare that they have no competing interests.

## Authors' contributions

TM and TH carried out most of the experiments in this study. KT carried out the immunostaining experiments. TI carried out some electrophysiological experiments. ES made and maintained EP_1_- and IP-deficient mice, and participated in the interpretation of data. YS and SN participated in experimental design and discussion. TT carried out some biochemical experiments. MT contributed to all aspects of the study and wrote the manuscript.
